# Fc receptor-like 5 and anti-CD20 treatment response in granulomatosis with polyangiitis and microscopic polyangiitis

**DOI:** 10.1172/jci.insight.136180

**Published:** 2020-09-17

**Authors:** Kasia Owczarczyk, Matthew D. Cascino, Cecile Holweg, Gaik W. Tew, Ward Ortmann, Timothy Behrens, Thomas Schindler, Carol A. Langford, E. William St. Clair, Peter A. Merkel, Robert Spiera, Philip Seo, Cees G.M. Kallenberg, Ulrich Specks, Noha Lim, John Stone, Paul Brunetta, Marco Prunotto, the RAVE-ITN Research Group

**Affiliations:** 1 Department of Cancer Imaging, King’s College London, London, United Kingdom.; 2 Genentech, South San Francisco, California, USA.; 3 Hoffmann – La Roche, Basel, Switzerland.; 4 Cleveland Clinic, Cleveland, Ohio, USA.; 5 Duke University Medical Center, Durham, North Carolina, USA.; 6 University of Pennsylvania, Philadelphia, Pennsylvania, USA.; 7 Hospital for Special Surgery, New York, New York, USA.; 8 Johns Hopkins University, Baltimore, Maryland, USA.; 9 University Medical Center Groningen, Groningen, Netherlands.; 10 Mayo Clinic College of Medicine and Science, Rochester, Minnesota, USA.; 11 Immune Tolerance Network (ITN), Seattle, Washington, USA.; 12 Massachusetts General Hospital, Boston, Massachusetts, USA.; 13 School of Pharmaceutical Sciences, University of Geneva, Geneva, Switzerland.; 14 The RAVE-ITN Research Group is detailed in Supplemental Acknowledgments.

**Keywords:** Autoimmunity, Clinical Trials, Vasculitis

## Abstract

**BACKGROUND:**

Baseline expression of *FCRL5*, a marker of naive and memory B cells, was shown to predict response to rituximab (RTX) in rheumatoid arthritis. This study investigated baseline expression of *FCRL5* as a potential biomarker of clinical response to RTX in granulomatosis with polyangiitis (GPA) and microscopic polyangiitis (MPA).

**METHODS:**

A previously validated quantitative PCR–based (qPCR-based) platform was used to assess *FCRL5* expression in patients with GPA/MPA (RAVE trial, NCT00104299).

**RESULTS:**

Baseline *FCRL5* expression was significantly higher in patients achieving complete remission (CR) at 6, 12, and 18 months, independent of other clinical and serological variables, among those randomized to RTX but not cyclophosphamide-azathioprine (CYC/AZA). Patients with baseline *FCRL5* expression ≥ 0.01 expression units (termed *FCRL5*^hi^) exhibited significantly higher CR rates at 6, 12, and 18 months as compared with *FCRL5*^lo^ subjects (84% versus 57% [*P* = 0.016], 68% versus 40% [*P* = 0.02], and 68% versus 29% [*P* = 0.0009], respectively).

**CONCLUSION:**

Our data taken together suggest that *FCRL5* is a biomarker of B cell lineage associated with increased achievement and maintenance of complete remission among patients treated with RTX and warrant further investigation in a prospective manner.

**FUNDING:**

The analysis for this study was funded by Genentech Inc.

## Abstract 2

FcRL5 is a biomarker of B cell lineage associated with maintenance of complete remission among patients treated with rituximab in granulomatosis with polyangiitis and microscopic polyangiitis.

## Introduction

Granulomatosis with polyangiitis (GPA) and microscopic polyangiitis (MPA) are small-vessel vasculitides that are characterized by the presence of antineutrophil cytoplasmic autoantibodies (ANCA). These autoantibodies activate neutrophils and monocytes, resulting in damage to the vascular endothelium (1). B cell depletion through treatment with rituximab (RTX), an anti-CD20 monoclonal antibody, was shown to be noninferior to a cyclophosphamide-azathioprine (CYC/AZA) regimen for the induction of remission in GPA/MPA in the RAVE trial (NCT00104299) (2). A total of 64% of the patients in the RTX group, as compared with 53% of the patients in the CYC/AZA group, had complete remission (CR) at 6 months. At 12 and 18 months, 48% and 39%, respectively, of the patients in the RTX group had maintained CR, as compared with 39% and 33%, respectively, in the comparison (3).

The RAVE study also showed that patients with relapsing disease (versus a new diagnosis) and those who were proteinase 3–ANCA^+^ (PR3–ANCA^+^) at baseline achieved complete response more frequently when treated with RTX compared with those patients without these characteristics. In contrast, there was no observed difference in response by treatment group among patients who were positive for antimyeloperoxidase (anti-MPO) ANCA (4). Interestingly, PR3-ANCA–positivity and relapsing disease at presentation are variables associated with highest risk of relapse (3, 5).

In our previous work, we developed a quantitative PCR–based (qPCR-based) platform to identify B cell lineage biomarkers of clinical response to RTX in rheumatoid arthritis (6). A subgroup of treated patients characterized by a combination of elevated baseline mRNA levels of *IgJ* (a marker for antibody secreting plasmablasts) and reduced levels of Fc receptor-like 5 (*FCRL5*), preferentially expressed on naive and memory B cells, predicted nonresponse to RTX in inadequate responders to anti-TNF therapy.

FCRL5, also known as CD307, is encoded by the immunoglobulin superfamily receptor translocation-associated 2 (*IRTA2*) gene and was originally cloned from myeloma cell lines. FCRL5 contains 9 extracellular Ig domains, as well as 2 immunoreceptor tyrosine–based inhibitory motifs (ITIM) and one presumed immunoreceptor tyrosine–based activation motif (ITAM) in its cytoplasmic tail. Using a chimeric receptor containing the cytoplasmic tail of FCRL5, cross-linking FCRL5 and the B cell receptor (BCR) was shown to recruit SHP-1 to the 2 ITIM motifs of FCRL5, resulting in reduced BCR-induced calcium mobilization and protein tyrosine phosphorylation (7). FCRL5 binds intact IgG via a complex mechanism; therefore, immune complexes may link FCRL5 to the BCR, potentially blocking B cell activation similarly to the inhibitory FcγRIIB or CD22 (8).

Intriguingly, *FCRL5* was found to be overexpressed in tissue-like memory B cells based on microarray analysis (9, 10). FCRL5^+^ cells were found enriched on CD21^−/lo^ CD27^+^IgM^+^ marginal zone–like B cells in patients with hepatitis C virus–related mixed cyoglobulinemia vasculitis (HCV-MC), but not in healthy donors (11); feature T-bet expression; and may be enriched during chronic antigenemia. Consistent with this, *FCRL5* mRNA and cell surface protein expression required prolonged BCR stimulation and de novo protein synthesis (12). FCRL5 was also found to be upregulated in circulating “atypical” memory B cells, which are associated with exposure to Plasmodium falciparum and may represent dysfunctional or exhausted B cells with downmodulated BCR signaling and reduced capacity to produce antibody and to undergo recall responses (13).

We investigated *FCRL5* expression levels in patients from the RAVE trial with the goal of determining whether *FCRL5* mRNA expression at baseline could serve as a predictive biomarker for achieving CR.

## Results

### Baseline FCRL5 expression in responders versus nonresponders.

**Figure 1:**
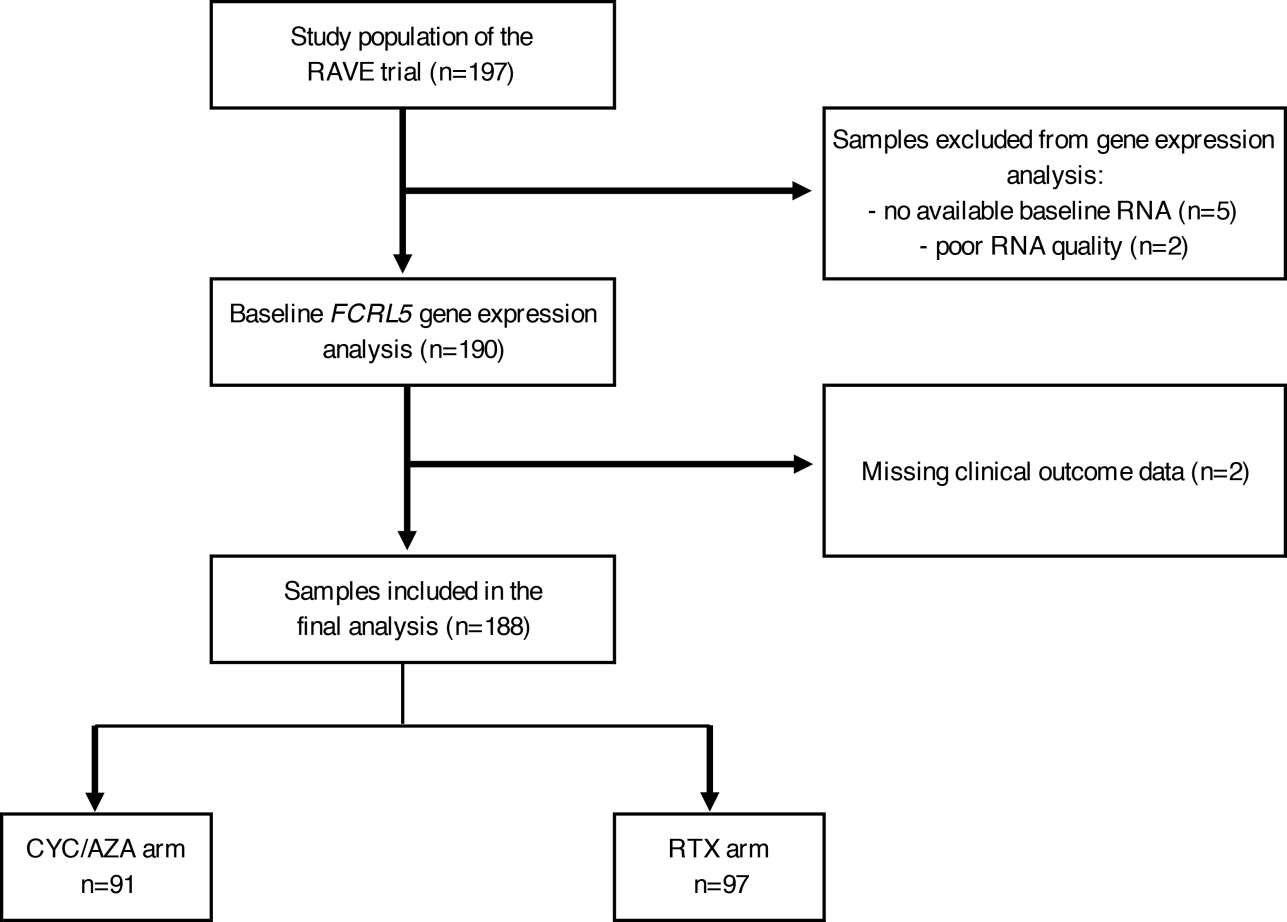
Sample processing flowchart.

*FCRL5* mRNA gene expression analysis was successfully carried out in 190 of 197 study subjects and matched with clinical data in 188 subjects, 97 in the RTX arm, and 91 in the CYC/AZA arm (Figure 1). Flow cytometry data were available for a subset of patients (*n* = 168; 86 in RTX arm and 82 in CYC/AZA arm).

The proportion of patients achieving CR at 6 months in this patient cohort was 64% in the RTX arm versus 54% in the CYC/AZA arm, consistent with response rates observed in the RAVE trial population.

**Figure 2:**
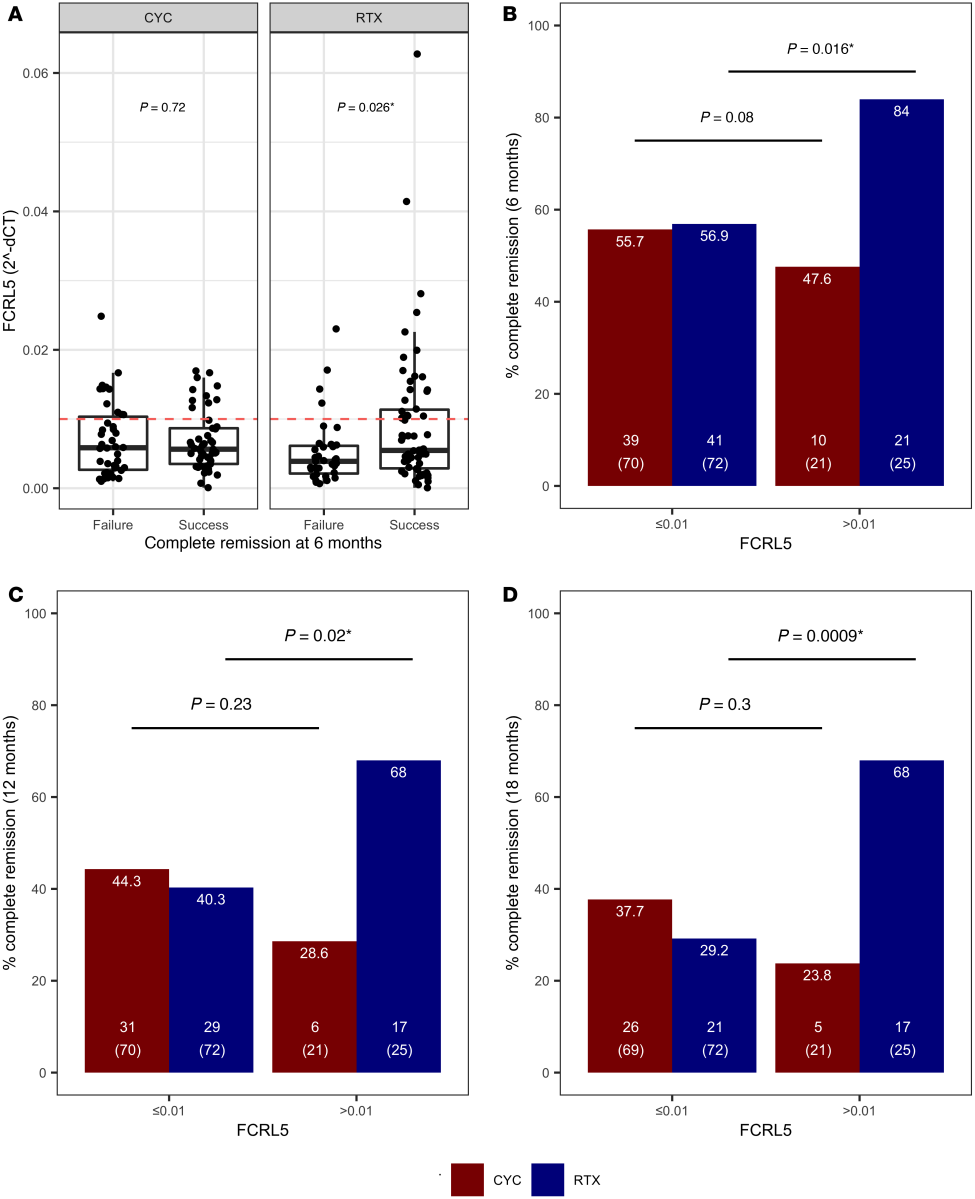
Validation of *FCRL5* mRNA gene expression biomarker in patients in the RAVE trial. (**A**) Baseline *FCRL5* mRNA levels assayed by qPCR in whole blood were compared in patients who achieved and failed to achieve complete remission at 6 months in both the RTX (*n* = 62 and *n* = 35, respectively) and the CYC arm (*n* = 49 and *n* = 42, respectively). Median and interquartile range shown as box plot; whiskers represent IQR. (**B–D**) Identified biomarker threshold (*FCRL5* ≥ 0.01 versus *FCRL5* < 0.01) was tested in baseline mRNA samples from patients in the RAVE trial as a predictor of complete remission at 6 months (**B**), 12 months (**C**), and 18 months (**D**), in subjects treated with RTX (blue bars) versus CYC (red bars). The number on the top of the bars in **B–D** denotes percentage remission rate in each subgroup, the number in brackets refers to the total number of subjects in each respective subgroup, and the number above it refers to the number of remitters. Wilcoxon rank-sum *P* value and Fisher’s exact *P* values shown in **A** and **B–D**, respectively. *P < 0.05.

In the RTX arm, mean baseline *FCRL5* level was significantly higher in patients who went on to achieve CR at 6 months as compared with these who did not (median 0.005 expression units [range 0.003–0.012] versus 0.004 [range 0.002–0.006]; *P* = 0.026) (Figure 2A). There was no difference in baseline *FCRL5* expression in responders versus nonresponders in the CYC/AZA arm.

### Response rates to RTX at 6, 12, and 18 months in the FCRL5^hi^ (FCRL5 ≥ 0.01) and FCRL5^lo^ (FCRL5 < 0.01) subgroups.

A threshold sensitivity analysis identified a threshold of 0.01 *FCRL5* expression units to be the most discriminatory for the 6-month response to RTX. Application of this threshold resulted in a significant enrichment for responders in the *FCRL5*^hi^ subgroup, in the RTX but not the CYC/AZA arm. In the RTX arm, baseline *FCRL5* expression was associated with CR at 6 months (*FCRL5*^hi^ 84% versus *FCRL5*^lo^ 57%, *P* = 0.016) (Figure 2B). In contrast, there was no difference in CR in the CYC/AZA arm (*FCRL5*^hi^ 48% versus *FCRL5*^lo^ 56%, *P* = 0.8). In both arms, *FCRL5*^hi^ subgroup represented roughly 25% of patients (25 of 97 in the RTX arm and 22 of 92 in the CYC/AZA arm).

Similar associations were identified at 12 months in the RTX arm (*FCRL5*^hi^ 68% versus *FCRL5*^lo^ 40%, *P* = 0.02) and the CYC/AZA arm (*FCRL5*^hi^ 29% versus *FCRL5*^lo^ 44%, not significant) (Figure 2C).

The rate of CR at 18 months in the RTX arm was only 29% in the *FCRL5*^lo^ subset (*P* = 0.0009) and 47% in all patients. In the CYC/AZA arm, these percentage rates were 38% and 34%, respectively (Figure 2D). These results and a summary of *FCRL5* levels by CR are shown in Table 1.

Examining the baseline characteristics between *FCRL5*^hi^ versus *FCRL5*^lo^ patients, we observed that a significantly lower proportion of *FCRL5*^hi^ patients were GPA (65%) and PR3^+^ (52%) as compared with *FCRL5*^lo^ where 81% and 73% of patients were GPA (*P* = 0.03) and PR3^+^ (*P* = 0.01), respectively (Table 2). Such trends were not observed in CYC/AZA patients stratified by *FCRL5* and CR (Supplemental Table 1; supplemental material available online with this article; https://doi.org/10.1172/jci.insight.136180DS1). Interestingly, a significantly lower proportion of RTX *FCRL5*^hi^ patients who achieved CR were PR3^+^ (48%) as compared with 78% in *FCRL5*^lo^ subgroup (Supplemental Table 2). No other differences were observed.

### Association of FCRL5 expression with peripheral B cell subtypes.

At baseline, *FCRL5* expression was positively correlated with the frequency of CD19^+^ cells (Spearman’s ρ = 0.46), specifically with the frequency of activated naive B cells (CD19^+^CD27^–^CD86^+^ [Spearman’s ρ = 0.47] and CD19^+^CD27^–^HLA-DR^+^ [Spearman’s ρ = 0.47]). There were no significant correlations with baseline CD20^+^ B cells, nonactivated and CD95-expressing memory cells, plasma, or germinal center founder cells. No correlation was observed with any of the T or myeloid cell populations.

In a random forest model, activated naive and memory B cell subsets were the most important predictors of *FCRL5* expression at baseline (CD19^+^CD27^–^HLA-DR percent mean squared error [MSE], 8.15; CD19^+^CD27^–^CD86^+^, 6.63; IGD^+^CD27^+^CD19^+^IGM^+^, 6.23; CD19^+^HLA-DR^+^, 5.84; and CD19^+^CD38^–^IgD^+^CD23^+^, 5.51) (Table 3).

**Figure 3:**
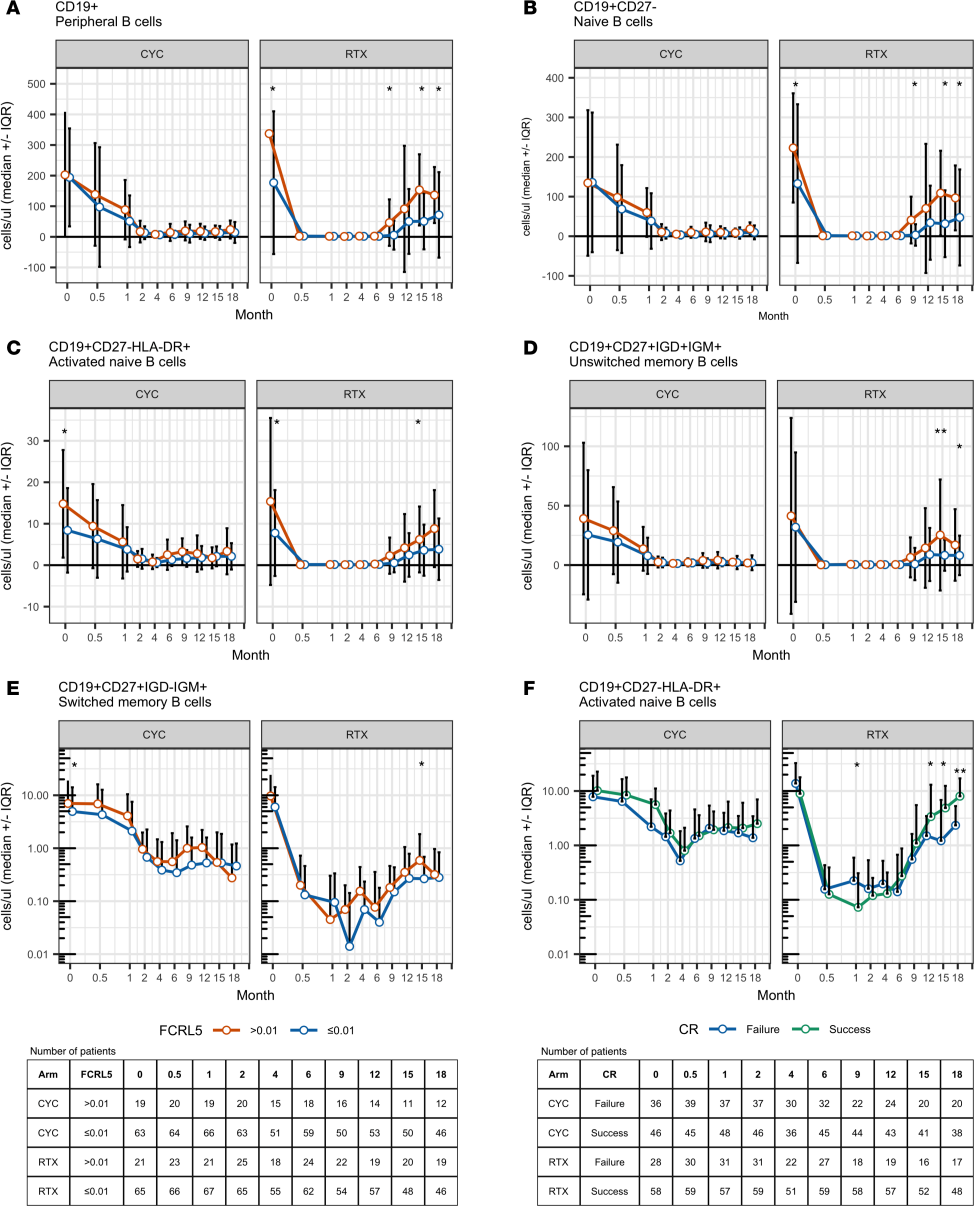
Longitudinal flow cytometry data. (**A–E**) patients have been stratified based on baseline *FCRL5* expression. (**F**) Subjects have been stratified based on complete remission at month 6. The numbers of patients with available data at each visit are shown in the tables. Wilcoxon rank-sum *P* values are as follows: *P < 0.05; **P < 0.01. CR, complete remission.

The effect of induction treatment on peripheral blood B cell subsets was similar in both treatment groups regardless of baseline *FCRL5* status (Figure 3). During the follow-up period after induction, particularly in the RTX group, many of the examined B cell subsets repopulated more rapidly among *FCRL5*^hi^ patients compared with *FCRL5*^lo^ patients. The difference in median values achieved statistical significance at several time points after month 6. When treated with RTX but not CYC/AZA, *FCRL5*^hi^ subjects repopulated with significantly higher CD19^+^ counts (Figure 3A), naive B cells (CD19^+^CD27^–^) (Figure 3B), activated naive B cells (CD19^+^CD27^–^HLA-DR^+^) (Figure 3C), nonswitched memory B cells (Figure 3D), and switched memory B cells (Figure 3E).

Of note, RTX subjects who failed to achieve CR at 6 months had higher activated naive B cells at the 1-month time point (Figure 3F). There was no difference in the percentage of the CD19^+^ B cells or the aforementioned B cell subsets throughout the induction period between patients who achieved or failed to achieve CR at 6 months in either the RTX or CYC/AZA arms (not shown).

### Univariate and multivariate analyses adjusting for clinical and serological parameters.

In univariate logistic regression analysis, *FCRL5* expression was significantly associated with CR at 18 months in the RTX arm (*P* = 0.016), and there was a trend toward achieving CR at 6 and 12 months. This trend at 6 months became significant in the multivariate analysis after adjusting for PR3-ANCA status, new onset versus relapsing disease status, and baseline Birmingham Vasculitis Activity Score for Wegener’s Granulomatosis (BVAS/WG) score (Table 4).

We found an association between log-transformed *FCRL5* as a continuous variable and achievement of CR at months 6 and 18. This implies direct correlation of response with *FCRL5* expression level, with an increase in *FCRL5* throughout the range of baseline values being associated with an increasing probability of CR in the RTX group. In contrast, there was no association between CR achievement and *FCRL5* levels in the CYC/AZA arm at any *FCRL5* level. There was sufficient separation between the RTX and CYC/AZA curves at the highest *FCRL5* levels to suggest a treatment benefit of RTX over CYC/AZA in this population. This effect nearly reaches statistical significance with respect to the primary endpoint (CR at 6 months; *P* = 0.087) despite being significantly underpowered for the analysis.

## Discussion

ANCA-associated vasculitis is a rare and life-threatening autoimmune disease associated with B cell hyperactivity, the presence of autoantibodies, and elevated BAFF levels (14), suggesting that B cells play a critical role in the pathogenesis of this disease. Recent trials have begun to identify targeted immunotherapies that represent new options compared with conventional CYC (2). PR3-ANCA^+^ and relapsing disease subgroups of patients in RAVE appear to receive greater benefit from RTX compared with CYC/AZA, providing clinicians with information that might impact therapeutic choices. Our analysis is important because it demonstrates that a baseline B cell marker in GPA and MPA is associated with increased achievement of CR among patients treated with RTX and may be associated with a prolonged duration of clinical remission following a single cycle of RTX therapy.

In RAVE, patients who achieved CR off glucocorticoids at 6 months in the RTX arm had significantly higher baseline *FCRL5* expression. *FCRL5* expression at baseline seems to predict CR at 6, 12, and 18 months in patients treated with RTX but not those treated with CYC/AZA. Furthermore, *FCRL5* expression appears to have a predictive value independent of other response-predictive factors such as type of vasculitis, serology, or presence of relapsed disease in a multivariate analysis.

While both RTX and CYC/AZA are peripheral blood B cell–depleting agents, our paper’s findings may relate to the greater depletion of CD20^+^ B cells seen with RTX. Baseline *FCRL5* expression in the peripheral blood of patients with GPA or MPA is correlated with the frequency of memory B cells — specifically, nonswitched IgD^+^IgM^+^ B cells — and, to a lesser extent, naive B cells. Although *FCRL5* was reported to be associated with switched memory B cells in healthy individuals (9), we observed only a weak correlation between *FCRL5* and switched IgD^–^IgM^+^ B cells in this study, potentially due to the differences in the population of the subjects or the possibility that *FCRL5* was enriched only in CD27^–^ atypical memory B cells, which were not quantified in this study. Despite higher total naive and memory B cell numbers at baseline, RTX *FCRL5*^hi^ patients had a similar extent of B cell depletion during the induction period compared with *FCRL5*^lo^ patients. The significance of B cell repletion in *FCRL5*^hi^ patients at later time points will require further investigation to understand the potential impact on relapse or PR3-ANCA status.

This analysis has several strengths. It represents an unbiased selection of available samples from the RAVE study, one of the largest, extensively sampled, blinded placebo-controlled interventional trials in GPA or MPA. The proportion of *FCRL5*^hi^ patients is approximately 25% of the baseline cohort in RAVE, making these findings relevant to a subfraction of patients. Taken together, these results are an intriguing step in the direction of precision medicine for these patients but are purely hypothesis generating. These findings should be replicated in additional studies of anti-CD20 agents or CYC in the treatment of these severely ill patients in need of CR.

One weakness of our analysis is that the sampling used for it was incomplete, and missing samples could have skewed or altered the analysis in an unknown manner. However, the number of unavailable samples was low (5%), and the trial outcomes of the subset included in our analysis mirrored that of the entire cohort. Another limitation of the study is that we do not have direct confirmation of *FCRL5* surface expression on B cells. It would be important for future studies to develop correlations between mRNA levels and surface expression in similar cohorts of clinically active GPA and MPA patients. A third weakness of the analysis is that further combinations of potentially predictive biomarkers such as B cell depletion status or ANCA status (3) were not evaluated together. Other groups have evaluated a Granularity Index from the RAVE publicly available database (15). Our analysis introduces a new and biologically relevant biomarker for consideration.

In conclusion, there is increasing interest in the identification of potentially novel response biomarkers in ANCA-associated vasculitis (15) that might aid therapeutic selection as new choices are made available to patients. RAVE is a NIAID-sponsored, ITN-supported open-source database that is available to a global community of researchers interested in these diseases. Truly useful biomarkers in many immunologic diseases are relatively rare. Our study findings of a subset of participants with higher baseline *FCRL5* expression could identify a subgroup of patients that may achieve a sustained benefit of RTX compared with CYC.

## Methods

Supplemental Methods are available online with this article.

### Study design.

The RAVE trial (NCT00104299) was a multicenter, randomized, double-blind noninferiority trial that compared RTX administered at a dose of 375 mg/m^2^ of body surface area once a week for 4 weeks followed by placebo with CYC administered at a dose of 2 mg/kg for 3–6 months followed by AZA at a dose of 2 mg/kg for 12–15 months for a total of 18 months of therapy. It enrolled ANCA^+^ patients with GPA and MPA who met criteria for severe disease (BVAS/WG > 3). Patients were randomly assigned in a 1:1 fashion to either RTX or CYC followed by AZA. Details of the RAVE trial design have been published elsewhere (2).

### Clinical assessments and outcome measures.

Study visits occurred weekly over the first 4 weeks of therapy, followed by visits at months 2, 4, and 6. Disease activity was assessed using the BVAS/WG instrument (16). The primary outcome measure for this study was CR at 6 months (BVAS/WG of 0 with a glucocorticoid dose of 0, which was the primary endpoint in the original RAVE study analysis; ref. 2). Secondary endpoints included remission maintained through 12 and 18 months.

### Sample collection and gene expression analysis.

Whole blood sample collection for gene expression analysis occurred at screening (visit 1) according to the trial protocol (2).

The samples were stored at the ITN core facility and transferred to Genentech for analysis. The methodology of mRNA gene expression analysis of candidate B cell markers has been previously published (6).

In brief, RNA was extracted from whole blood using PAXgene blood RNA kits (QIAGEN) according to manufacturer’s protocol. The amount and quality of RNA extracted were assessed using both NanoDrop (Thermo Fisher Scientific) and Agilent 2100 Bioanalyzer technologies, respectively.

qPCR was performed using the ABI 7900HT instrument (Applied Biosystems). We used a commercially available, inventoried ABI assay encoding *FCRL5* splice variant — *FCRL5/IRTA2C* (Hs01070204_m1; Applied Biosystems) — shown in preliminary experiments to be expressed predominantly on naive and mature B cells, as opposed to *IRTA2A* and *IRTA2B,* which are expressed predominantly in bone marrow plasma cells. Expression of each gene was measured in duplicate in each experiment, and the average of the replicates was normalized to human housekeeping gene *GAPDH* (Hs99999905_m1; Applied Biosystems) to generate a ΔCt value for each gene. Data were analyzed using BioMark Gene Expression Data Analysis software (Fluidigm Corporation) to obtain Ct values. Expression data were then calculated as relative abundance, using the formula 2^–ΔCt^.

### Flow cytometry data.

Flow cytometry data were provided by the RAVE-ITN Group. This data set is available for download from the Immunology Database and Analysis ITN Portal and TrialShare public repositories (https://www.immunetolerance.org/researchers/trialshare).

Flow cytometry analysis was carried out at predefined time points in the RAVE trial — at screening, at week 2, and at months 1, 2, 4, 6, 9, 12, 15, and 18 following enrollment — and was carried out using standardized protocols described in detail elsewhere (15).

### Statistics.

The Biostatistics Team from the study sponsor (NIAID) provided raw, deidentified, individual-level patient data. These data sets are accessible to readers through TrialShare, a publicly accessible website developed and managed by the ITN (https://www.immunetolerance.org/researchers/trialshare). Selected clinical data were transferred from clinical trial databases into a customized Valicert database designed to facilitate analysis. Data analysis was performed using JMP (SAS), GraphPad Prism (GraphPad), and R statistical package (version 3.5.3) (www.R-project.org).

Differences in baseline *FCRL5* expression between responders and nonresponders (expressed as CR at 6, 12, and 18 months) in the RTX and CYC/AZA arms with respect to the linearly transformed values were measured using the nonparametric Wilcoxon rank-sum test.

A threshold sensitivity method described elsewhere (6) was applied to baseline RNA samples in order to identify a *FCRL5* biomarker threshold that enriched for a control arm–corrected CR (at 6 months) to RTX. This threshold once established was then used in binary comparisons in order to compare outcomes in patients termed *FCRL5*^lo^ and *FCRL5*^hi^. Differences in baseline variables (demographic, clinical, serological, and molecular data) — as well as in the proportion of patients achieving CR at 6, 12, and 18 months for the *FCRL5*^lo^ and *FCRL5*^hi^ patient subsets — were calculated, and nominal *P* values were determined. Continuous variables were compared using the Wilcoxon rank-sum test. For categorical variables, 2 separate contingency tables, 1 for the experimental arm (RTX) and 1 for the control arm (CYC/AZA), were created to compare the proportion of responders in the *FCRL5*^lo^ and *FCRL5*^hi^ subsets, and *P* values were calculated using Fisher’s exact test.

Spearman’s rank correlation (ρ) was used as a nonparametric test of association between flow cytometry cell populations at the screening visit and baseline FCRL5 expression. To identify flow cytometry populations that were independently correlated with *FCRL5* expression, a random forest decision tree algorithm was used to rank variables based on their predictive importance (expressed as proportion of MSE [%MSE]).

Differences in FACS populations between *FCRL5* subgroups (high ≥ 0.01 versus low < 0.01) at each time point were compared using Wilcoxon rank-sum test.

Logistic regression was used to investigate the relationship between *FCRL5* and other baseline clinical characteristics. In univariate analysis, each characteristic was considered individually in separate logistic regression models. For multivariate analysis, all characteristics were included in a single logistic regression model to see if effects remained after adjustment for other factors. Statistical significance threshold was set at *P* < 0.05. No correction was applied to account for multiple testing.

### Study approval.

The RAVE trial was approved by the IRB at each participating site (2). Written informed consent was obtained from all patients before inclusion in the study.

## Author contributions

KO, GWT, MP, and PB wrote and edited the manuscript. TS, MDC, WO, TB, CH, CAL, and GWT generated and analyzed the data contained in the present manuscript. JS and US contributed critical thinking and revision of the manuscript. PAM, MDC, and CH reviewed the manuscript and provided comments. All authors contributed to data interpretation.

## Supplementary Material

Supplemental data

## Figures and Tables

**Table 1:**
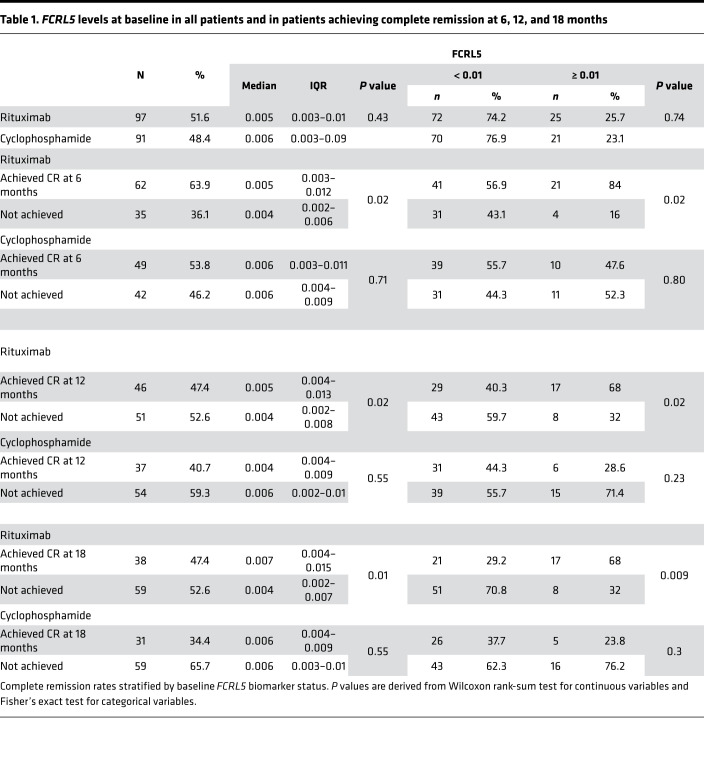
*FCRL5* levels at baseline in all patients and in patients achieving complete remission at 6, 12, and 18 months

**Table 2:**
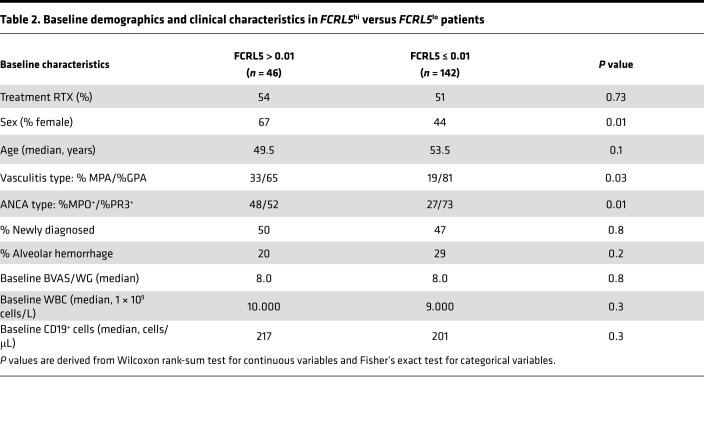
Baseline demographics and clinical characteristics in *FCRL5*^hi^ versus *FCRL5*^lo^ patients

**Table 3:**
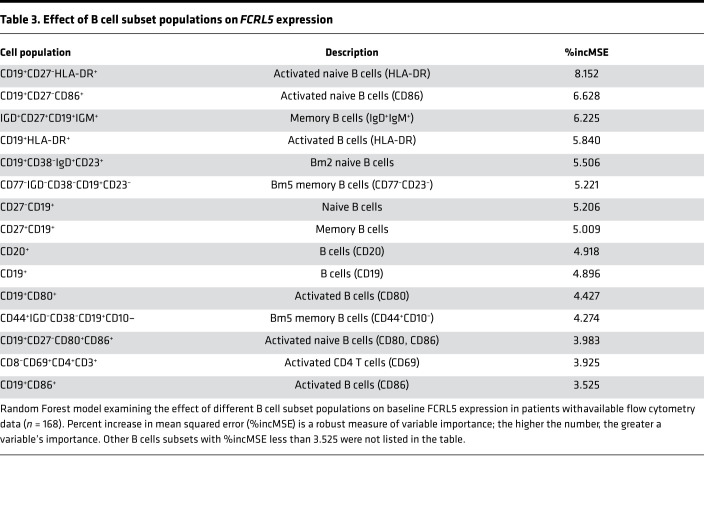
Effect of B cell subset populations on *FCRL5* expression

**Table 4:**
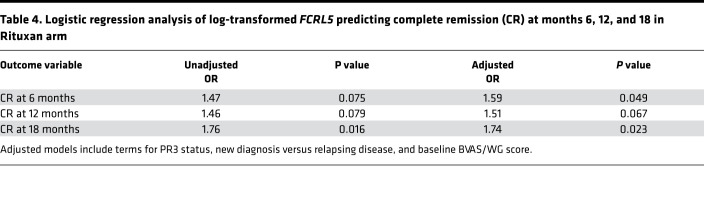
Logistic regression analysis of log-transformed *FCRL5* predicting complete remission (CR) at months 6, 12, and 18 in Rituxan arm

## Version 1 08/25/2020

In-Press Preview

## Version 2 09/17/2020

Electronic publication

## References

[1] Jennette JC, Falk RJ (2014). Pathogenesis of antineutrophil cytoplasmic autoantibody-mediated disease. Nat Rev Rheumatol.

[2] Stone JH (2010). Rituximab versus cyclophosphamide for ANCA-associated vasculitis. N Engl J Med.

[3] Specks U (2013). Efficacy of remission-induction regimens for ANCA-associated vasculitis. N Engl J Med.

[4] Unizony S (2016). Clinical outcomes of treatment of anti-neutrophil cytoplasmic antibody (ANCA)-associated vasculitis based on ANCA type. Ann Rheum Dis.

[5] Hogan SL (2005). Predictors of relapse and treatment resistance in antineutrophil cytoplasmic antibody-associated small-vessel vasculitis. Ann Intern Med.

[6] Owczarczyk K (2011). A plasmablast biomarker for nonresponse to antibody therapy to CD20 in rheumatoid arthritis. Sci Transl Med.

[7] Haga CL, Ehrhardt GR, Boohaker RJ, Davis RS, Cooper MD (2007). Fc receptor-like 5 inhibits B cell activation via SHP-1 tyrosine phosphatase recruitment. Proc Natl Acad Sci U S A.

[8] Franco A (2013). Human Fc receptor-like 5 binds intact IgG via mechanisms distinct from those of Fc receptors. J Immunol.

[9] Li H, Borrego F, Nagata S, Tolnay M (2016). Fc Receptor-like 5 Expression Distinguishes Two Distinct Subsets of Human Circulating Tissue-like Memory B Cells. J Immunol.

[10] Chang LY, Li Y, Kaplan DE (2017). Hepatitis C viraemia reversibly maintains subset of antigen-specific T-bet+ tissue-like memory B cells. J Viral Hepat.

[11] Terrier B (2014). CD21(-/low) marginal zone B cells highly express Fc receptor-like 5 protein and are killed by anti-Fc receptor-like 5 immunotoxins in hepatitis C virus-associated mixed cryoglobulinemia vasculitis. Arthritis Rheumatol.

[12] Damdinsuren B, Dement-Brown J, Li H, Tolnay M (2016). B cell receptor induced Fc receptor-like 5 expression is mediated by multiple signaling pathways converging on NF-κB and NFAT. Mol Immunol.

[13] Sullivan RT (2015). FCRL5 Delineates Functionally Impaired Memory B Cells Associated with Plasmodium falciparum Exposure. PLoS Pathog.

[14] Krumbholz M, Specks U, Wick M, Kalled SL, Jenne D, Meinl E (2005). BAFF is elevated in serum of patients with Wegener’s granulomatosis. J Autoimmun.

[15] Nasrallah M (2015). Reanalysis of the Rituximab in ANCA-Associated Vasculitis trial identifies granulocyte subsets as a novel early marker of successful treatment. Arthritis Res Ther.

[16] Stone JH (2001). A disease-specific activity index for Wegener’s granulomatosis: modification of the Birmingham Vasculitis Activity Score. International Network for the Study of the Systemic Vasculitides (INSSYS). Arthritis Rheum.

