# Supplementary material for: Fc receptor-like 5 and anti-CD20 treatment response in granulomatosis with polyangiitis and microscopic polyangiitis
Source: JCI Insight. 2020 Sep 17;5(18):e136180. doi: 10.1172/jci.insight.136180 (PMC7526555; doi:10.1172/jci.insight.136180)
Supplement: Supplemental data [file jciinsight-5-136180-s054.pdf]

Fc receptor-like 5 and anti-CD20 treatment response in granulomatosis with polyangiitis and microscopic polyangiitis

Kasia Owczarczyk[1], Matthew D. Cascino[2], Cecile Holweg[2], Gaik W. Tew[2], Ward Ortmann[2], Timothy Behrens[2], Thomas Schindler[3], Carol A. Langford[4], E. William St. Clair[5], Peter A. Merkel[6], Robert Spiera[7], Philip Seo[8], Cees G.M. Kallenberg[9], Ulrich Specks[10]†, Noha Lim[11]†, John Stone[12]†, Paul Brunetta[2*, ¥] and Marco Prunotto[2,13*,#]

Supplementary Data

Supplementary Methods

Study

Details of the RAVE clinical trial (ClinicalTrials.gov identifier NCT00104299) have been published elsewhere (2). The trial was approved by the institutional review board at each participating site. Participants and site staff were blinded as to participant treatment assignment. This trial was monitored by the sponsor (National Institute of Allergy and Infectious Diseases) and adverse events were reviewed by the Data Safety Monitoring Board.

Supplementary Results

Table S1. Baseline demographics and clinical characteristics in *FCRL5*^{high} versus *FCRL5*^{low} subgroups stratified by complete remission at 6 months in CYC/AZA patients.

**CYC/AZA only**	***FCRL5* >0.01**			***FCRL5* ≤0.01**			
**Complete Remission at 6 months**	**Success n=10**	**Failure n=11**	**P value#**	**Success n=39**	**Failure n=31**	**P value#**	**P value^**
Sex (% female)	70	55	0.66	41	39	1.0	0.16
Age (median, years)	53	48	0.32	52	52	0.63	0.45
Vasculitis type: % MPA/ %GPA	50/50	10/90	0.063	21/79	13/87	0.53	0.10
ANCA type: %MPO+/%PR3+	60/40	18/82	0.081	33/67	19/81	0.28	0.16
% Newly diagnosed	70	27	0.086	56	35	0.097	0.50
% Alveolar hemorrhage	40	18	0.36	18	32	0.26	0.20
Baseline BVAS/WG (median)	8.5	6.0	0.19	8.0	7.0	0.68	0.38
Baseline WBC (median, 10^9 cells/l)	7.6	9.8	0.35	10.0	8.1	0.022*	0.037*
Baseline CD19+ cells (median, cells/ul)	194	219	0.97	185	177	0.88	0.41

P values are derived from Wilcoxon rank-sum test for continuous variables and Fisher's exact test for categorical variables. #p-value compared success versus failure subgroups; ^p-value compared success subgroups between *FCRL5*^{hi} versus *FCRL5*^{low} .

Table S2. Baseline demographics and clinical characteristics in *FCRL5*^{high} versus *FCRL5*^{low} subgroups stratified by complete remission at 6 months in RTX patients.

**RTX only**	***FCRL5* >0.01**			***FCRL5* ≤0.01**			
**Complete Remission at 6 months**	**Success n=21**	**Failure n=4**	**P value#**	**Success n=41**	**Failure n=31**	**P value#**	**P value^**
Sex (% female)	67	100	0.30	49	45	0.81	0.28
Age (median, years)	47	62	0.58	56	58	0.91	0.083
Vasculitis type: % MPA/ %GPA	33/62	50/50	0.68	22/78	20/80	1.0	0.16
ANCA type: %MPO+/%PR3+	52/48	75/25	0.60	22/78	32/68	0.42	0.022*
% Newly diagnosed	48	75	0.59	44	52	0.63	0.79
% Alveolar hemorrhage	14	0	1.0	32	35	0.80	0.22
Baseline BVAS/WG (median)	9.0	6.5	0.061	7.0	9.0	0.37	0.21
Baseline WBC (median, 10^9 cells/l)	11.3	9.7	0.58	10.3	11.3	0.19	0.69
Baseline CD19+ cells (median, cells/ul)	217	311	0.89	261	269	0.54	0.88

P values are derived from Wilcoxon rank-sum test for continuous variables and Fisher's exact test for categorical variables. #p-value compared success versus failure subgroups; ^p-value compared success subgroups between *FCRL5*^{hi} versus *FCRL5*^{low} . #p-value compared success versus failure subgroups; ^p-value compared success subgroups between *FCRL5*^{hi} versus *FCRL5*^{low}.

Conflict of interests: All authors have completed the ICMJE disclosure form. KO, CAL, EWSC, PAM, RS, PS, CGMK, US, NL, JS declare no financial support from, or relationship with any organization that may have an interest, for the submitted work. Dr. Merkel reports personal fees from AbbVie, grants and personal fees from AstraZeneca, personal fees from Biogen, grants and personal fees from Boeringher-Ingelheim, grants and personal fees from Bristol-Myers Squibb, grants and personal fees from Celgene, grants and personal fees from ChemoCentryx, personal fees from CSL Behring, personal fees from Forbius, grants and personal fees from Genentech/Roche, grants and personal fees from Genzyme/Sanofi, grants and personal fees from GlaxoSmithKline, grants and personal fees from InflaRx, personal fees from Insmed, personal fees from Jannsen, personal fees from Kiniksa, grants from Kypha, personal fees from Magenta, personal fees from Novartis, personal fees from Pfizer, personal fees from Sparrow, grants from TerumoBCT, other from UpToDate, personal fees from Talaris, outside the submitted work. MDC, CH, GWT, WO, TB and PB are employees of Genentech Inc. TC and MP are employees of Roche.

Acknowledgements:

Members of the RAVE-ITN Research Group are as follows: *Protocol Cochairs* — U. Specks (Mayo Clinic), J.H. Stone (Massachusetts General Hospital); *Mayo Clinic* — U. Specks, S.R. Ytterberg, F.C. Fervenza, K.A. Keogh, T. Peikert, J.M. Golbin, L. Klein, K. Mieras, C. Beinhorn, S. Fisher, M.L. Clawson, S. Bendel, A.M. Hummel (Mayo Clinic Eisenberg Research Pharmacy); *Boston University* — P.A. Merkel, E.Y. Kissin, P.A. Monach, M.R. Clark-Cotton, C.A. McAlear, J.L. Pettit, M.B. Sutton, R.L. Widom, G.A. Farina, M.J. DiMarzio, S.P. Johnson, A. Schiller Patel; *Johns Hopkins University* — P. Seo, J.H. Stone, D. Hellmann, D. Geetha, A. Saleh, P. Wung, L.P. Sejismundo, C. Humphrey, M. Marriott, Y. Goldsborough, A. Pinachos, K. Gauss, L. King; *Cleveland Clinic Foundation* — C.A. Langford, G.S. Hoffman, R.A. Hajj-Ali, J.J. Carey, E.S. Molloy, C.L. Koening, D. Bork, T.M. Clark, K.A. Tuthill, T. Markle, J. Petrich; *Hospital for Special Surgery* — R. Spiera, D.R. Alpert, S.J. DiMartino, J.K. Gordon, N.K. Moskowitz, K.A. Kirou, J. Samuels, S.A. Kloiber, E. Julevic, M. O'Donohue, A. Patel; *University of Groningen* — C.G.M. Kallenberg, C. Stegeman, P. Rasker, K. Mulder, P. Limburg, J. Kosterink; *Duke University* — E.W. St. Clair, N.B. Allen, E. Scarlett, M. Tochacek; *University of Alabama–Birmingham* — A. Turkiewicz, B. Fessler, W. Chatham, A. Turner; Coordinating Centers: *Rho* — D. Ikle, D. Weitzenkamp, W. Wu, T. D'Lugin, C. Jacob; *National Institute of Allergy and Infectious Diseases* — L. Webber, L. Ding, S. Adah; *Immune Tolerance Network* — N.K. Tchao, M. Mueller, K. Bourcier, A. Asare, V. Seyfert-Margolis, P. Tosta, N.B. Skeeter, C.L. Anderson, A.N. Archampong.
